# Research on the Mechanical Properties of Concrete under Low Temperatures

**DOI:** 10.3390/ma17081882

**Published:** 2024-04-19

**Authors:** Xiangyi Li, Lihui Qin, Lina Guo, Yan Li

**Affiliations:** 1 School of Water Resources and Civil Engineering, Northeast Agricultural University, Harbin 150030, China; s210101045@neau.edu.cn (X.L.); guolina1900@sina.com (L.G.); 2 School of Transportation Science and Engineering, Harbin Institute of Technology, Harbin 150030, China; liyan_2007@126.com

**Keywords:** low temperature, concrete, strength level, uniaxial compressive strength characters, constitutive

## Abstract

As construction projects in cold regions continue to increase, it has become necessary to understand the performance of concrete at low temperatures. Conducting uniaxial compressive tests on non-standard prismatic concrete specimens under low-temperature conditions and analyzing the test results allows for a comprehensive understanding of the strength variations of concrete with different strength grades at temperatures of 20 °C, 0 °C, −20 °C, −30 °C, and −40 °C. When the temperature decreases from 20 °C to 0 °C, the compressive strength of the specimens decreases, while the elastic modulus and peak strain increase. As the temperature continues to decrease, the compressive strength of the specimens increases, the elastic modulus continues to grow, and the peak strain decreases. The rising segments of the curves can be fitted using a cubic polynomial, and as the temperature decreases further, the parameters of the fitting curve gradually decrease. For concrete, being the most widely used material in the construction field, understanding its performance in low-temperature environments has become a significant research topic in the field of materials engineering and construction.

## 1. Introduction

Concrete is the most widely used material in the field of hydraulic engineering [1]. With the vigorous development of construction in Western China, more and more projects are inevitably being carried out in cold regions. Currently, in northern China and other regions, the winter temperatures are relatively low. In the cold environment, the water in the pores of concrete materials will freeze and expand, causing damage to the surrounding concrete materials, resulting in significant changes to the stress state. This leads to differences in the mechanical properties and bearing capacity of the concrete materials [2]. Due to the long winter season, large-scale project construction, extended construction period, tight schedule, and the need to carry out diversion and flood control projects using the dry season, it is necessary to carry out construction work in winter. Studying and exploring the mechanical properties of concrete at low temperatures is of great theoretical and engineering significance for the construction of hydraulic engineering projects in cold regions.

A substantial amount of experimental research on the mechanical properties of concrete under low temperatures has been conducted by researchers both domestically and internationally. The compressive strength of concrete at low temperatures is influenced by factors such as admixtures, water content, strength grade, and curing methods. concrete manifests much improved compressive strength at low temperatures [3]. Dahmani [3,4] has demonstrated that as the temperature decreases, the compressive strength of concrete increases accordingly. Bairagi [5] found that the strength of concrete materials increases under low temperatures, but it decreases when returning from low temperatures to room temperature. Much higher compressive strengths were obtained at the cryogenic temperature for both NC and UHPFRC compared with those at the ambient and recovered ambient temperatures [6]. Xie et al. [5,7] have concluded that there is a basic linear relationship between the elastic modulus of concrete and temperature. Lee [8] has found that as the temperature decreases, the elastic modulus of concrete increases, but at a rate lower than its compressive strength. The compressive strength, Poisson’s ratio, Young’s elastic modulus and thermal conductivity increase with decreasing temperatures [9]. Browne [10] believes that the increase in strength at low temperatures is related to the water content; the higher the water content, the more significant the strength enhancement. Krstulovic-Opara [11] has conducted a systematic and detailed study on the performance of concrete in ultra-low temperature environments, indicating that the degree of improvement in the mechanical properties of concrete materials under ultra-low temperatures is closely related to the water content. Longarini [12] indicates that using fly ash in the mix design for concrete leads to a significant improvement in fresh concrete properties. Zhang [13] indicate that fly ash concrete has higher strength under different low temperatures compared to concrete strength at room temperature, especially at −30 °C, followed by −40 °C, −10 °C, and −20 °C. Shi Xudong [14] have proven that the relative increase in the compressive strength of concrete is positively correlated with its strength grade. The higher the strength grade, the greater the strength increment; however, this increment becomes gradual and insignificant when the concrete strength grade reaches C50 and above [15]. München [16] points out that the strength of concrete is related to the curing time, with a slow increase in strength at 7 days and a 28% increase in strength at 28 days.

The concrete constitutive relationship curve is the most fundamental constitutive relationship and serves as the primary basis for studying concrete deformation and load-bearing performance, with extensive research conducted by many experts and scholars in this field [17]. Yu [18] have established a modified D-P constitutive model based on temperature and strain rate. Xu [19] proposed a concrete computational constitutive model based on dynamic loads. Li [20] conducted axial compressive strength tests and utilized a dual-parameter expression proposed by Guo Zhenhai to represent the ascending and descending segments of the stress–strain curve of concrete at different temperatures. Su [21] conducted research on the damage and failure mechanisms of materials subjected to impact loads using a mesoscopic model of concrete composed of aggregates, mortar, ITZs, and ice particles. Masad [22] conducted a low-temperature experimental study on concrete for liquefied natural gas (LNG) storage tanks and established an ABAQUS concrete damage model that can predict the damage of concrete under low temperatures. Gong [23] has simulated the static mechanical behaviors of concrete under different situations are simulated based on a two-dimensional discrete model rigid body spring method.

The (GB 51081-2015) [24] stipulates the mechanical performance requirements for concrete below −40 °C. However, the Mohe area in Heilongjiang Province of China experiences an average winter temperature of −30 °C, while temperatures in the northern cold regions of China range from 0 °C to −40 °C. Therefore, the (GB 51081-2015) is not applicable to winter construction in China. Applying performance theories from normal temperature conditions to low-temperature construction may result in concrete specimens failing to meet design strength requirements, leading to potential structural damage [25]. Using concrete performance at normal temperatures for designs in low-temperature conditions poses certain issues [26]. Current research has mainly focused on the performance of low-temperature concrete at ultra-low temperatures (T ≤ −100 °C), while there is little evaluation of the performance of concrete at ordinary low temperatures (−40 °C ≤ T ≤ 20 °C). Thus, this study conducts uniaxial compression tests at five temperatures—20 °C, 0 °C, −20 °C, −30 °C, and −40 °C (with 20 °C as the normal temperature reference condition) to offer guidance for winter construction in China. Research and exploration of the mechanical properties of concrete at normal and low temperatures have important theoretical and engineering implications for the field of materials science and engineering.

## 2. Materials and Methods

### 2.1. Experimental Design

The present study designs test specimen dimensions in accordance with the dimensions described in the (GB/T 50081-2019) [27], while considering the requirements of the experimental equipment. The experimental design employed four strength grades of C30, C35, C40, and C50, with specimens of dimensions 100 mm × 100 mm × 300 mm in a prismatic shape. A total of 60 specimens were produced and divided into 20 groups of three specimens each, based on the four strength grades and five temperature grades. The main factors considered were the impact of temperature parameters and strength grade on concrete strength, with corresponding temperature points of 20 °C, 0 °C, −20 °C, −30 °C, and −40 °C.

Considering the comprehensive factors of the(GB/T 50082-2010) [28] and the (JGJ55-2011) [29], the mix proportion of concrete is calculated and designed. The mix proportions and number of specimens are shown in Table 1.

### 2.2. Specimen Fabrication

The properties and concentrations of raw materials affect the related technical properties of concrete. The raw materials used in this experiment meet the technical specifications required by the norms, and the following are the materials used in the experiment:

(1) Cement: P.O 42.5 Ordinary Portland cement produced by Harbin Yongxing Cement Products Co., Ltd., (Harbin, China).
(2) Sand: with an absorption rate of 14.68%, a moisture content of 3.72%, a bulk density of 1386 kg/m^3^, an apparent density of 2287 kg/m^3^, and fineness modulus of 2.3.
(3) Aggregate: Comprises two sizes, 5 mm to 9 mm and 9 mm to 16 mm, that were mixed in the ratio of 4:6 by mass. With an absorption rate of 1.01%, a moisture content of 0.52%, a bulk density of 1453 kg/m^3^, and an apparent density of 2558.5 kg/m^3^.
(4) Water for mixing and curing concrete: ordinary tap water in (Harbin, China).

Before production, appropriate mineral oil is applied inside each mold. The raw materials are weighed according to the ratio, placed into a pre-wetted mixer, and mixed evenly. The mixture is rapidly transferred into the mold, vibrated on a vibrating table for 1 min, and then the surface is leveled. The test blocks are covered with plastic wrap for one day of indoor curing, and after setting, the molds are removed and placed in a standard curing room with a relative humidity of >95% and a temperature of 20 ± 2 °C for further curing. After 28 days of curing, the specimens are transferred to a refrigerator for freezing at the designated temperature. During the cooling process, due to the thermal inert properties of concrete, different regions of the test blocks exhibit varying temperatures, leading to an uneven temperature distribution.

Liu [30] pointed out that, in the case of a cooling rate of T = 1 °C/min, it takes 5 to 6 h to achieve a uniform temperature distribution across the section if a constant temperature state is desired. Therefore, in this experiment, the cooling time to the designated temperature and constant temperature is set to 48 h to ensure a uniform distribution of interface temperature. To reduce experimental errors, all test blocks of the same proportions are produced in one batch, and the mixture is taken from the same mixing process.

The specimens of the same strength grade in the experiment were cast from the same batch, with the same mixture ratio, so it can be assumed that the specimens of the same strength grade have similar moisture contents during the analysis process. The residues obtained after subjecting the specimens to a high-temperature oven at 200 °C for a duration of 72 h were measured to yield moisture contents of 2.61%, 2.54%, 2.49%, and 2.32% for C30, C35, C40, and C50 concrete, respectively.

### 2.3. Experimental Methods

The experiment utilizes the WAW-1000 microcomputer-controlled electro-hydraulic servo universal testing machine, produced by Shanghai Hualong Testing Instrument Co., Ltd., (Shanghai, China). Concrete deformation is measured using the displacement of the testing machine itself. Following the loading method under normal temperature conditions, with displacement control applied at a rate of 1.5 mm/min until specimen failure occurs, and the maximum load is recorded. All specimens are subjected to uniaxial compression tests to obtain information such as strength, elastic modulus, and peak strain under low-temperature conditions for different strength grades of specimens. The experimental procedures are as follows:

1. Test specimens are rapidly removed from the low-temperature environmental chamber, the surface moisture is dried, and an external inspection is conducted to ensure no apparent defects are present.
2. Before conducting the pressure test, the bearing surface is identified and undergoes abrasive treatment to ensure that the loading plate can adhere closely to the bearing surface and is parallel to the casting surface. The test specimen is placed at the center of the loading plate of the testing machine to ensure that the center of the specimen bears the pressure. Subsequently, the testing machine is activated, and the ball joint seat is adjusted when the upper pressing plate is about to contact the specimen to achieve a state of balance upon contact, thus minimizing eccentricity as much as possible. It is particularly noted that the casting surface must not serve as the bearing surface.
3. The loading rate is to be continuous and uniform, with a rate of 1.5 mm/min employed. When the specimen approaches failure, adjustment of the throttle is ceased, and the test continues until the specimen fails, at which point the load is recorded.

## 3. Results and Discussion

### 3.1. Experimental Phenomena

(1) Apparent Condition of Specimens After Low-Temperature Treatment.

As the temperature continues to decrease, a thin layer of frost forms on the surface of the specimens, with no noticeable change in the overall appearance or color. The specimens remain intact, without the formation of any visible new cracks on the surface, and there are no signs of chipping, peeling, or other forms of deterioration.

(2) Characteristics of Low-Temperature Compressive Failure.

**Figure 1:**
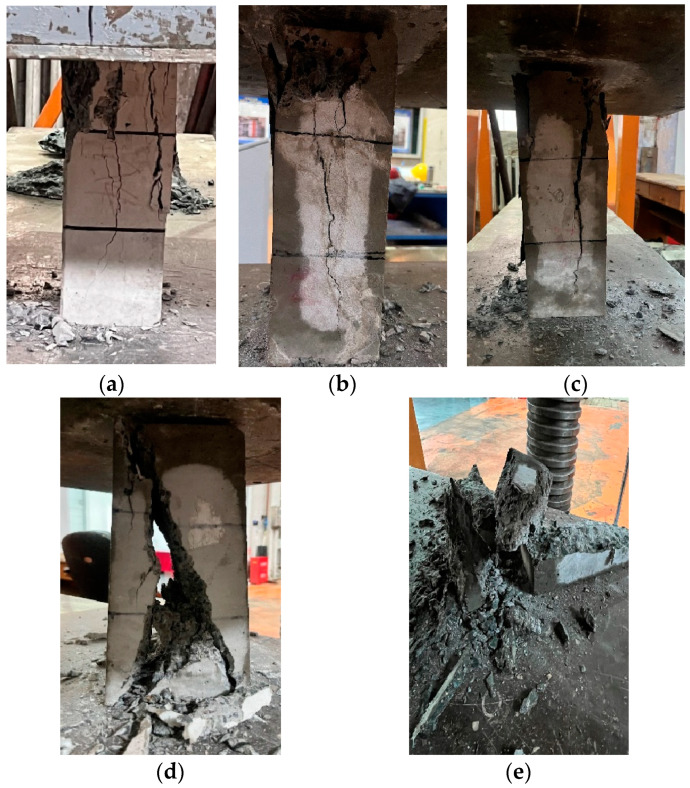
Concrete compression behavior at low temperatures: (**a**) 20 °C, (**b**) 0 °C, (**c**) −20 °C, (**d**) −30 °C, (**e**) −40 °C.

At the initial application of load, there may be some minor cracks, pores, or other compressive voids that exhibit slight closure or enlargement due to stress concentration at a particular crack, yet no visible cracks are formed in the overall concrete specimen [25]. As the load approaches 0.8 f_c_ to 0.9 f_c_, a microscopic visible crack gradually appears in the central region of the specimen, extending from top to bottom along the load direction. As the load is continuously applied, some concrete spalling occurs at the top of the specimen, accompanied by splitting sounds, followed by the flaking off of the corners in a sheet-like manner. Short longitudinal cracks emerge on the specimen, which are parallel to the direction of force. The concrete in the middle region begins to laterally expand, and the cracks in the middle become wider and deeper, gradually forming diagonal cracks that eventually penetrate the entire cross-section. As can be seen from Figure 1, the changes in the specimen under different temperatures are depicted. As the temperature decreases, cracks in the specimens will continue to widen, generating more fragments. The extent of damage to the specimens varies significantly at different temperatures. At 20 °C, as shown in Figure 1a, the specimen remains relatively intact, with two long cracks in the middle and some damage to the upper edges, but still maintaining its original contour. As the temperature decreases, as depicted in Figure 1b at 0 °C and Figure 1c at −20 °C, the cracks in the specimens further deepen and expand, with extensive damage to the upper edges and more fragments generated during loading. However, at −30 °C, as shown in Figure 1d, the cracks in the specimen penetrate the entire block, splitting it into several pieces. By the time the specimen reaches −40 °C, as seen in Figure 1e, it is severely damaged, shattered into fragments, with its original shape unrecognizable.

During the loading process, the curve continues to rise as the load is applied, reaching peak stress and then rapidly dropping, indicating a rapid loss of bearing capacity. It is challenging to capture the descending segment of the concrete due to the stiffness limitations of the testing machine being used, as the machine stiffness fails to meet the requirements.

As the temperature decreases, the fragmented pieces obtained after fracturing gradually become smaller and tend towards a more standardized conical shape. The damage at the top surface of the specimen becomes increasingly severe, leading to almost all of them eventually turning into fragmented pieces without a complete top surface [30]. When test blocks of the same strength grade are loaded to the same load, as the temperature of the specimens decreases, the cracks generated in the blocks become deeper and wider, and the fracture pattern becomes more distinct. Due to the discreteness of concrete, different specimens exhibit different failure characteristics in experiments. In some specimens, detachment occurs at the upper end first, followed by the gradual development of cracks. In others, tiny cracks appear directly, which then deepen and extend, resulting in detachment at the upper surface. Some specimens also show detachment in the middle section. Observations of the damaged specimens even reveal the rare occurrence of coarse aggregate fracture [31].

### 3.2. Analysis of Test Results

The uniaxial compressive stress–strain curve of concrete forms the basis of the concrete constitutive relationship, and is crucial for understanding the load-bearing capacity and deformation behavior of concrete structures. Through analyzing parameters like elastic modulus, strength, and peak strain, the constitutive relationship is investigated. Axial compression tests are being carried out to assess the compressive strength of concrete specimens at different strength levels under low temperatures. The average compressive strength, average elastic modulus, and average peak strain of the test blocks are shown in Table 2.

#### 3.2.1. Compressive Strength

**Figure 2:**
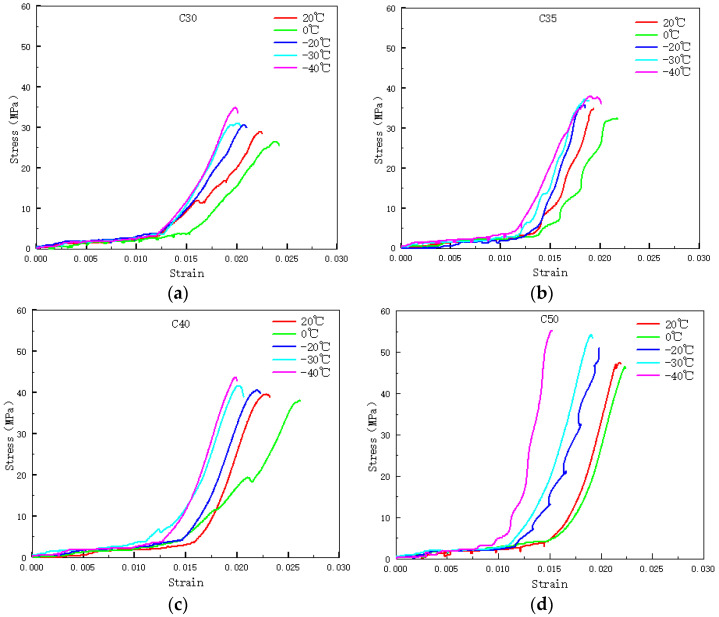
Stress–strain curves of concrete prisms at different temperatures: (**a**) C30, (**b**) C35, (**c**) C40, (**d**) C50.

Within the same condition, the curve representing 0 °C is positioned at the far right, signifying the flattest curve, with the specimen having minimal stress and maximum strain. As the specimen undergoes a temperature decrease from 20 °C to 0 °C, the stress diminishes and the strain intensifies. Beginning at 0 °C, with the temperature steadily declining, the specimen’s stress progressively amplifies, whereas the strain gradually diminishes, leading to a sharper curve. Figure 2 indicates that, under all four conditions a–d, this law is satisfied. Therefore, under the same condition, specimens experience a decrease in stress and an increase in strain as they go from 20 °C to 0 °C. Starting from 0 °C, as the temperature continues to decrease, the stress continues to increase and the strain to decrease. Under the same conditions, but in the same condition, the change in specimen strength is solely dependent on temperature and not on its strength grade.

#### 3.2.2. Strength Grade

**Figure 3:**
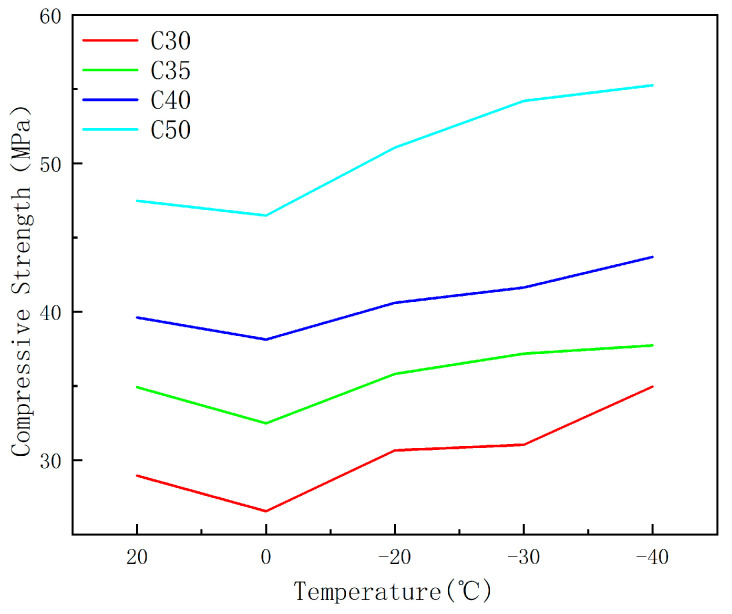
The effect of temperature on compressive strength.

**Figure 4:**
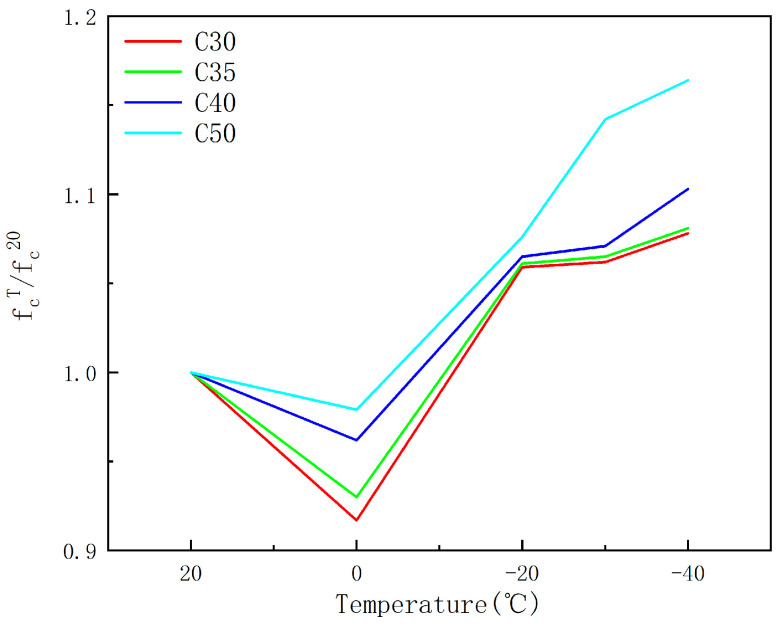
The relationship between relative strength and temperature.

Figure 2 illustrates that as the transition occurs from C30 to C50, the strength grades of the concrete specimens incrementally elevate, leading to a transformation in the curves from gradual to abrupt. The strain values for varying conditions align closely from 0 to 0.015, subsequent to which the curves diverge. A positive correlation is observed between the steepness of the curves and the strength grades, with higher strength grade conditions exhibiting greater slope values. Concurrently, as temperatures descend continuously, the strain overlap region diminishes, shifting from approximately 0 to 0.015 to roughly 0 to 0.0125, indicating a consistent reduction in strain. Figure 3 demonstrates that under the same temperature, and without considering other factors such as water content, the curves decrease sequentially with increasing strength grades, indicating that concrete specimens with higher strength grades exhibit greater strength. The relative concrete compressive strength at different temperatures, referred to as the strength relative value hereinafter, is expressed in terms of the ratio of the compressive strength of concrete prisms at a specific temperature (f_c_^T^) to that at 20 °C (f_c_^20^). The relationship between relative strength and temperature are shown in Figure 4.

Figure 3 reveals that the strength growth rate of C30 concrete specimens relative to the reference temperature of 20 °C is as follows: −8.3%, 5.9%, 6.2%, and 7.8%. For C35, the growth rates are: −7.0%, 6.1%, 6.5%, and 8.1%. For C40, the rates are: −3.8%, 6.5%, 7.1%, and 10.3%. And for C50, the rates are: −2.1%, 7.6%, 14.2%, and 16.4%. It is observed that with increasing strength grades, the relative compressive strength of concrete increases more significantly. In Figure 4, it can be observed that the relative strength values for C50 concrete are higher, and the higher the strength class of the test blocks, the greater their strength relative values. Furthermore, at a constant temperature, concrete blocks with a higher strength class exhibit greater compressive strength. This is in agreement with the conclusion of Shi [14]. Existing research has suggested that the strength of concrete reaches a certain temperature at which it no longer continues to increase, rather than decreasing with temperature reduction [32]. Different strength grades of concrete have different internal structures, with varying amounts and distributions of water and pores. Berner [33] suggests that ice directly contributes to stress reduction by filling internal pores. The strength grade and moisture content of concrete are interrelated and cannot be analyzed separately. Some scholars believe that the strength of concrete does not continuously increase with temperature reduction. Therefore, the above research indicates that the strength grade of concrete is closely related to its internal pore structure. Considering the strength grade alone cannot yield accurate results, as conclusions differ depending on the other variables involved. Therefore, it is necessary to take into account other variables to provide a more comprehensive and accurate conclusion.

#### 3.2.3. Elastic Modulus

**Figure 5:**
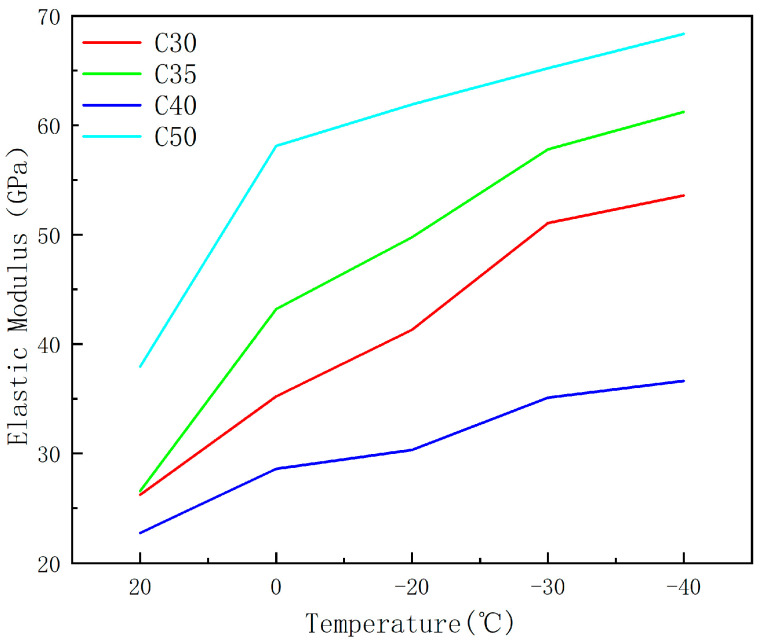
The effect of temperature on elastic modulus.

The elastic modulus of concrete, which affects its load-bearing capacity, stiffness of structural components, seismic resistance, and other properties, is one of the important indicators used to evaluate the various performance of concrete. Similar to compressive strength, the elastic modulus of concrete exhibits significant increases under low temperatures. In this study, the method adopted is to take the curve secant modulus in the range of 0.2 to 0.6 times the peak stress from the stress–strain curve in the ascending segment as the low-temperature elastic modulus of concrete. As shown in Figure 5, the elastic modulus of C30 concrete specimens relative to the reference temperature of 20 °C is as follows: 1.2%, 1.9%, 2.2%, and 2.6%. For C35, the increases are 1.4%, 1.9%, 2.2%, and 2.6%. For C40, the rates are 1.1%, 1.6%, 2.4%, and 2.7%. For C50, the rates are 1.5%, 2.0%, 2.2%, and 2.5%. The elastic modulus of concrete increases with temperature reduction under the same strength grade. The magnitude of the increase in elastic modulus does not appear to be significantly related to the strength grade, as evidenced by a comparison with Figure 3, where the growth in elastic modulus is found to be less pronounced than the growth in compressive strength. This phenomenon can be attributed to the hydrophilic nature of concrete, which consists of solid, liquid, and gaseous phases. Marshall [34] suggests that ice formed within the pores of the material affects the performance changes at low temperatures. During cold conditions, water within the concrete solidifies into ice, causing volume expansion and filling the internal voids of the specimen, thereby compacting pores and enhancing overall strength [20]. At the same temperature, specimens with higher strength grades exhibit larger elastic moduli. Monfore [35] and others have shown that the elastic moduli of ordinary concrete and lightweight aggregate concrete continuously increase from 0 °C to −100 °C, with the rate of increase in elastic modulus slowing down when the temperature drops below −100 °C. Lee [8] has demonstrated a continuous increase in the elastic modulus of concrete, following a trend similar to the increase in compressive strength, albeit at a much smaller rate.

#### 3.2.4. Peak Strain

As one of the important indicators of concrete’s mechanical properties and failure mechanisms, the analysis of its peak strain variations is beneficial for enhancing its safety and reliability. The peak strain of concrete reflects the material’s ductility, as a lower peak strain indicates poorer ductility, making it more susceptible to brittle failure [36]. Consistent with the behavior at room temperature, the peak strain of specimens at low temperatures also slightly increases with the improvement of strength grades.

**Figure 6:**
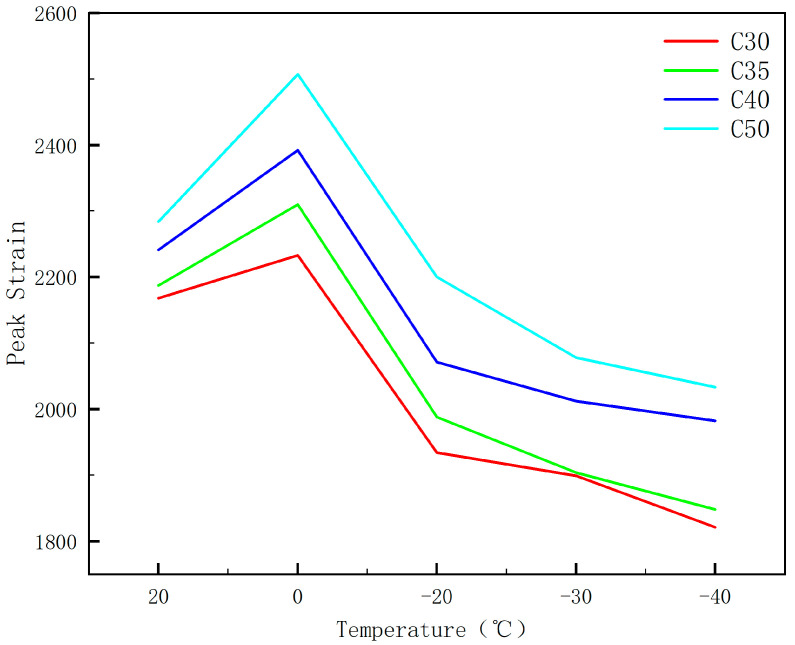
The effect of temperature on peak strain.

Figure 6 reveals that the peak strain of C30 concrete specimens exhibits a temperature-dependent growth rate, varying by 3.0%, −10.8%, −12.4%, and −16.0% compared to the standard temperature of 20 °C, respectively. For C35 concrete specimens, the respective growth rates are 5.6%, −9.1%, −12.9%, and −15.5%. C40 concrete specimens show increases of 6.7%, −7.6%, −10.2%, and −11.6%, while for C50 concrete specimens, the increases are 9.8%, −3.7%, 9.0%, and −11.0%. It is apparent that the peak strain of concrete specimens of the same strength class exhibits a distinct transition at 0 °C. Between 20 °C and 0 °C, the peak strain increases as the temperature decreases. Conversely, between 0 °C and −40 °C, the peak strain diminishes as the magnitude of negative temperatures increases. Additionally, as the strength class of the concrete improves, there is a slight yet noticeable enhancement in the peak strain. The information indicates that as the load is consistently applied, cracks are initially formed in the aggregate-cement gel under compression. These cracks gradually deepen and widen, with minor cracks also emerging within the cement gel. As the cracks continue to propagate, diagonal cracks ultimately spread across the entire interface, resulting in the rapid loss of load-bearing capacity in the test specimens.

### 3.3. Ontological Relationship

Currently, there are many models of concrete constitutive equations, such as using a unified expression for the ascending and descending portions of stress–strain curves: multiple, exponential, trigonometric, and rational equations, with few parameters and simple forms, which are easy to calculate. However, their curve shapes do not satisfy all the geometric characteristics of the experimental curves. In addition, the parameters of the unified equation for the upper and lower sections cannot be independently varied based on curve characteristics, and their values do not have specific numerical meanings, making it difficult to flexibly apply them [37].

A dimensionless stress–strain fitting expression was proposed by Guo of Tsinghua University [38] and others, with the mathematical expression as follows:

(1) $$y=ax+\left(3-2a\right)x^{2}+\left(a-2\right)x^{3},\;\;\;\;0<x<1$$

(2) $$y=\frac{x}{\alpha {\left(x-1\right)}^{2}+x},\;\;\;\;x>1$$

The *X*-axis represents the dimensionless stress, while the *Y*-axis represents the dimensionless strain. The parameter a in the ascending segment has a mechanical meaning of the ratio between the initial elastic modulus to the concrete secant modulus at the peak point. The parameter α in the descending segment is positively correlated with the absolute value of concrete stiffness. As α increases, the curve becomes steeper and approaches complete brittle failure [39].

Wang [40] has identified that the brittle failure of concrete specimens occurs after reaching peak strain due to the insufficient rigidity of the testing machine. During the loading process, the deformation of the testing machine increases with the load, accumulating significant elastic strain energy. When the load capacity of the specimen reaches its maximum and starts to decrease, the testing machine, as the applied stress decreases, quickly releases energy, applying a large additional compressive strain on the specimen, leading to the rapid failure of the concrete. This experiment only gathered stable data from the ascending segment. The curve fitting expression proposed by Guo and others for the ascending segment exhibits compliance with the geometric characteristics of the present experimental curves. The parameters of the upper and lower segments are independent of each other. The curve parameters a and α possess definite physical (geometric) significance and can be employed as quantitative indicators to analyze or compare the differences in mechanical properties among various concrete specimens. Therefore, the expression for the ascending segment (1) from Guozhenhai and others is adopted in this study.

**Figure 7:**
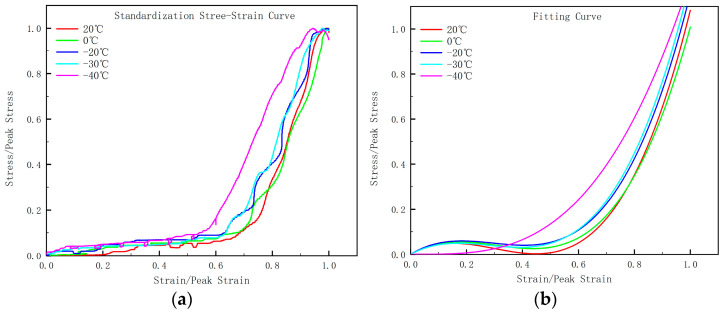
Standardization curve and fitting curve at different temperatures: (**a**) standardization stress–strain curve; (**b**) fitting curve.

The curves at various temperature points need to be standardized, which means transforming the horizontal axis into the ratio of stress to corresponding peak stress ε/ε_c_, and the vertical axis into the ratio of strain to peak strain σ/f_c_. After standardization, the curves are more concentrated compared to the original ones. The standardized stress–strain curves (as shown in Figure 7a) are fit according to Equation (1), and the resulting values of parameter a are summarized in Table 3.

From Table 3, under the same operating conditions, as the temperature decreases, the parameter a gradually decreases, while the elastic modulus and strength increase gradually. The parameter a show a fluctuating downward trend, indicating that the ratio of the initial elastic modulus of concrete to the peak secant modulus gradually decreases, and the stress–strain curve in the ascending segment tends to be more linear. Concrete with lower strength exhibits smaller deformation in the early stage, and strain development is faster after stress increases, resulting in a larger value of parameter a. The fitted curve for the entire range (taking C30 as an example) is shown in Figure 7.

From Figure 7, it can be observed that as the temperature decreases, the curve becomes steeper, meaning that the area under the curve is smaller, indicating lower plastic deformation, residual strength, faster failure speed, and a more brittle material [38]. Comparing Figure 7a,b, it is evident that the trend of the standardized stress–strain curve aligns closely with the fitted curve. The fitted curve is more centralized, and the fitting coefficient R^2^ values are as follows: 0.982, 0.990, 0.987, 0.991, 0.968. All fitting coefficients are above 0.95, demonstrating a good fitting effect.

## 4. Conclusions

As the progression of reform and development continues, an increasing number of construction and development projects are emerging in cold regions, such as those in Northwest China and the Tibetan Plateau. When these projects are initiated in cold areas, the challenges brought about by low temperatures become increasingly prominent. Under such conditions, the mechanical properties of concrete at low temperatures are particularly crucial, and a comprehensive understanding of these properties is essential to ensure the smooth progression of engineering projects. This article presents five conclusions for reference:

(1) Under the same mix ratio, the concrete strength decreases as the temperature decreases from 20 °C to 0 °C, and increases as the temperature decreases from 0 °C to −40 °C.
(2) At the same temperature, and without considering other factors such as moisture content, the stronger the concrete sample’s strength grade, the greater its strength will be. However, considering only the impact of strength grade cannot yield accurate results, as conclusions vary depending on the other variables. Therefore, it is necessary to include these other variables in the consideration scope to provide a more comprehensive and accurate conclusion.
(3) Similar to the trend of concrete strength, the elastic modulus of low-temperature concrete increases as the temperature decreases.
(4) Consistent with the behavior at room temperature, the peak strain of concrete increases slightly with the improvement in strength grade at low temperatures. Taking 0 °C as a dividing point, the peak strain increases with temperature decrease from 20 °C to 0 °C, and decreases with the increase of negative temperatures from 0 °C to −40 °C, although the amplitude of increase is smaller than that of strength. With the advancement of strength grade, the peak strain of concrete also experiences a slight increase.
(5) Under different temperatures, the stress–strain curve of concrete can be fitted by a cubic polynomial with variable parameters for the ascending portion. With the decrease in temperature, the parameters of the fitted curve for the ascending portion gradually decrease.

Contrary to the majority of studies that examine concrete properties at sub-zero temperatures, this paper focuses on the properties of concrete within the temperature range of 20 °C to −40 °C, providing more theoretical support for practical engineering construction. In the future, factors such as moisture content can be further introduced to comprehensively consider the influence of different strength grades of concrete on the strength of specimens and the tensile strength and durability of concrete at low temperatures. Meanwhile, further research should involve testing of 100 mm × 100 mm × 100 mm cubic specimens and cylindrical specimens with φ150 × 300 mm dimensions to explore the influence of different sizes on low-temperature concrete, in order to apply them to design programs.

## Figures and Tables

**Table 1:** Mix proportions and numbering of concrete with different strength levels.

Strength Grade	Composition (kg/m^3^)				Serial Number
	Cement	Water	Sand	Aggregate	
C30	24.60	14.76	41.21	73.31	A1~A15
C35	24.8	12.40	41.54	73.90	B1~B15
C40	25.4	11.43	42.55	75.69	C1~C15
C50	25.60	7.68	42.88	76.29	D1~D15

**Table 2:** Summary of test results.

Strength Grade	Temperature (°C)	Average Compressive Strength (MPa)	Average Elastic Modulus (GPa)	Average Peak Strain (×10^−6^)
C30	20	28.96	21.68	22.75
	0	26.56	21.94	23.43
	−20	30.67	22.09	20.29
	−30	30.76	22.16	19.93
	−40	31.22	22.25	19.11
C35	20	34.91	21.87	26.22
	0	32.47	22.18	27.69
	−20	37.04	22.29	24.10
	−30	37.18	22.36	22.84
	−40	37.74	22.44	22.16
C40	20	39.61	22.41	26.57
	0	38.10	22.66	28.35
	−20	42.18	22.77	24.55
	−30	42.43	22.95	23.86
	−40	43.69	23.02	23.49
C50	20	47.47	22.84	37.57
	0	46.47	23.18	41.25
	−20	51.08	23.30	36.18
	−30	54.21	23.34	34.19
	−40	55.26	23.42	33.44

**Table 3:** Summary of parameter a values.

Parametric	Strength Class	Temperature				
		20 °C	0 °C	−20 °C	−30 °C	−40 °C
a	C30	2.21	2.18	2.07	1.85	1.72
	C35	2.37	2.32	2.19	2.03	1.84
	C40	2.48	2.39	2.27	2.11	1.98
	C50	2.62	2.58	2.39	2.22	2.02

## Data Availability

Data are contained within the article.
